# The Similarities and Differences between the Effects of Testosterone and DHEA on the Innate and Adaptive Immune Response

**DOI:** 10.3390/biom12121768

**Published:** 2022-11-27

**Authors:** Fidel Orlando Buendía-González, Martha Legorreta-Herrera

**Affiliations:** 1 Laboratorio de Inmunología Molecular, Unidad de Investigación Química Computacional, Síntesis y Farmacología de Moléculas de Interés Biológico, Facultad de Estudios Superiores Zaragoza, Universidad Nacional Autónoma de México, Iztapalapa, Ciudad de México 09230, Mexico; 2 Posgrado en Ciencias Biológicas, Unidad de Posgrado, Edificio D, 1° Piso, Circuito de Posgrados, Ciudad Universitaria, Coyoacán, Ciudad de México 04510, Mexico

**Keywords:** testosterone, DHEA, DHT, orchidectomy, gonadectomy, dimorphism, male, androgen receptor (AR)

## Abstract

Androgens are steroids that modulate various processes in the body, ranging from reproduction, metabolism, and even immune response. The main androgens are testosterone, dihydrotestosterone (DHT) and dehydroepiandrosterone (DHEA). These steroids modulate the development and function of immune response cells. Androgens are generally attributed to immunosuppressive effects; however, this is not always the case. Variations in the concentrations of these hormones induce differences in the innate, humoral, and cell-mediated immune response, which is concentration dependent. The androgens at the highest concentration in the organism that bind to the androgen receptor (AR) are DHEA and testosterone. Therefore, in this work, we review the effects of DHEA and testosterone on the immune response. The main findings of this review are that DHEA and testosterone induce similar but also opposite effects on the immune response. Both steroids promote the activation of regulatory T cells, which suppresses the Th17-type response. However, while testosterone suppresses the inflammatory response, DHEA promotes it, and this modulation is important for understanding the involvement of androgens in infectious (bacterial, viral and parasitic) and autoimmune diseases, as well as in the sexual dimorphism that occurs in these diseases.

## 1. Introduction

Androgens are steroid hormones with immunoregulatory properties, which interact with many cells of the immune system, both innate and adaptive immunity. These hormones modulate different responses in lymphoid and nonlymphoid tissues after interacting with the androgen receptor (AR). In general, androgens possess immunosuppressive properties, which at least partly explains the increased susceptibility of males compared to females to a variety of parasitic, bacterial, and viral infections [1,2,3,4,5].

There is evidence that androgens modulate the immune system. Testosterone and dehydroepiandrosterone (DHEA) are the androgens with the highest concentrations. The concentration of androgens is age and sex dependent, which complicates understanding their role in the immune response [6,7,8]. Understanding this phenomenon will contribute to explaining the greater susceptibility of males to bacterial, parasitic, and viral infections. This work reviews the main mechanisms of action of androgens, as well as their effects on Toll-like receptors, immune response cells and pro- and anti-inflammatory cytokines.

## 2. Search Strategy and Screening Criteria

In this narrative review, the effects of androgens on the immune response are discussed. Emphasis is directed toward testosterone and DHEA, as they are the androgens in the highest concentration in humans. The discussion was based on a bibliographical review in PubMed covering articles written in English and published between 2000 and 2022, including important literature from the 1990s. The keywords used were “androgens”, “testosterone”, “DHEA”, “Dehydroepiandrosterone”, “DHT”, “hormone”, “orchiectomy”, “gonadectomy”, “dimorphism”, “males”, “androgen receptor”, “immune response” and “Infection”. All these words were combined with other keywords related to the immune response, such as cytokines, infections, components of the immune response, “Toll like receptors”, “innate immune response”, “adaptive immune response”, “dendritic cells”, “T cells”, “B cells”, “macrophage”, “Th1”, “Th2” and “Th17”. Clinical studies in humans and murine models and in vivo and in vitro studies were used as inclusion criteria. Only those reviews that contribute to defining a particular concept or a widely accepted phenomenon were cited.

## 3. Synthesis of Androgens Testosterone, DHT, Androstenedione and DHEA and Its Main Properties

**Figure 1:**
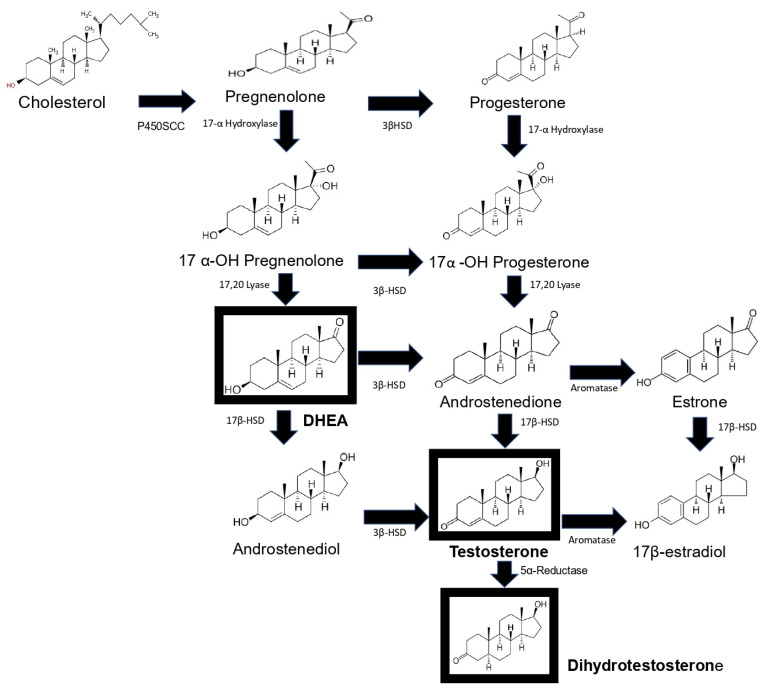
Schematic representation of androgen synthesis. Steroidogenesis starts with the cleavage of cholesterol by the P450SCS enzyme, which transforms it into pregnenolone, which is hydroxylated at carbon 17 by 17α-hydroxylase; the resulting product is the hormone DHEA. DHEA is converted to androstenediol by the enzyme 17β-HSD. This steroid and androstenedione are transformed to testosterone by the enzymes 3β-HSD and 17β-HSD, respectively. Dihydrotestosterone (DHT) is synthesized from testosterone. Finally, androstenedione is converted into estrone by the enzyme aromatase and estrone into 17β-estradiol by 17βHSD, and testosterone is transformed into 17β-estradiol by aromatase. The main sex hormones are highlighted in a box. This image based on the text described in [12].

Synthesis of androgens starts with cholesterol [9,10], which is converted to pregnenolone through the action of the enzyme P450scc. This steroid in turn is converted to 17-OH pregnenolone by the enzyme 17α-hydroxylase. 17-OH pregnenolone is transformed to DHEA by the enzyme 17,20 lyase, and DHEA is in turn sulfated (DHEA-S) by the enzyme sulfotransferase, which keeps it stable for longer. DHEA and DHEA-S are the androgens with the highest concentrations in human blood circulation [11]. DHEA is subsequently converted to androstenediol, and androstenedione is transformed into testosterone by the enzymes 17β-hydroxysteroid dehydrogenase (17βHSD) and 3β-hydroxysteroid dehydrogenase (3βHSD). Androstenediol and androstenedione are converted into testosterone by the enzymes 3βHSD and 17β-HSD. In addition, testosterone is transformed into DHT via the enzyme 5α-reductase [12]. Finally, testosterone and androstenedione can be transformed into estrogens by the enzyme aromatase. DHT is the only androgen that is not converted to estrogen. Androstenedione is transformed to estrone by the P450 aromatase and estrone to 17β-estradiol by the enzyme 17β-HSD. Testosterone can also be transformed into 17β-estradiol by P450aromatase [12]. Androgens testosterone and DHT possess a 17β-hydroxyl and a 3-oxo group, and the former and reduction of the latter result in the loss of biological activity [13] (Figure 1).

Androgens not only determine biological sex but also impact health and disease. The four androgens DHT, testosterone, androstenedione and DHEA act on many cells, including those of the immune system, and influence their function, maturation and susceptibility to damage by autoimmune processes [14]. The biological function of these steroids depends on their concentration, affinity and availability to interact with their receptors [15]. In addition, its concentration varies with sex and declines with age [6]. The potencies of androgens fluctuate; DHT is the most potent (300%), with testosterone (100%), androstenedione (10%) and DHEA (only 5%) [16]. In diseases such as rheumatoid arthritis, systemic lupus erythematosus, or multiple sclerosis, there is a marked sexual dimorphism; women are more susceptible than men. In contrast, in cancer, this pattern is reversed.

Testosterone is the most concentrated androgen in adult men, and DHT constitutes only 10% of the testosterone concentration. In men, 2% of testosterone is free, and 30% binds to sex hormone binding globulin (SHBG) with high affinity; the remaining testosterone binds with lower affinity to albumin and other proteins [17]. Interestingly, only free testosterone has biological activity [18]; therefore, the proteins that bind androgens modulate their action. On the other hand, DHEA binds with low affinity to the AR compared to testosterone or DHT. DHEA-S has high affinity for albumin and a long half-life, and it does not bind to AR [12]. DHEA also binds to estrogen receptors α and β (ERα and ERβ) [19]. Moreover, DHEA and DHEA-S act as ligands for G protein-coupled receptors and several nuclear receptors. Both steroids can modulate different signaling pathways [20]. The above explains the versatility and specificity of the functions that androgens play in the body.

## 4. Mechanisms of Action of AR-Dependent Androgens (Canonical Pathway)

**Figure 2:**
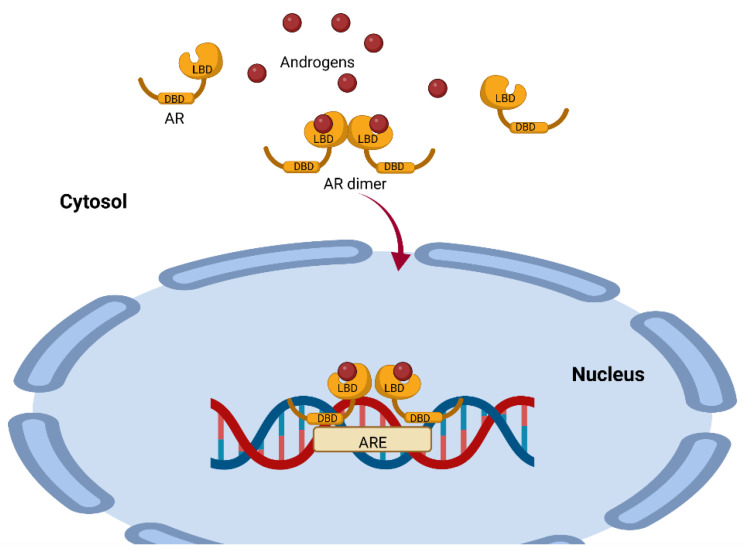
Representation of the mechanism of androgen action mediated by the androgen receptor. The AR has a highly conserved ligand-binding region (LBD) and a highly conserved DNA-binding region (DBD). Once the ligand binds to the AR, it dimerizes with another AR–ligand complex and translocates to the nucleus, where it binds to androgen response elements (AREs), modulating the expression of several genes. This image was based on the text of [28] and created using the BioRender.com software.

AR is a single-chain molecule present in the cytoplasm and is a ligand-dependent transcription factor. This receptor has three domains, the N-terminal domain (NTD), the DNA-binding domain (DBD) and the ligand-binding domain (LBD), which are all highly conserved (Figure 2) [21]. For steroid receptors to acquire the proper conformation that allows them to bind to their ligand, a highly ordered maturation process involving chaperones and cochaperones is required [22]. Some of the proteins involved include heat shock protein 90 (Hsp90), a 23 kDa chaperone and an FKBP52 protein containing a peptide with an Hsp90-binding TPR tetratricopeptide domain [23]. This complex is a positive regulator of the AR and for the glucocorticoid receptor GR [24]; the complex dissociates once the receptors have bound to its ligands (testosterone, DHT, androstenedione and DHEA) [25].

The AR–ligand complex dimerizes with another AR–ligand complex, undergoes phosphorylation and translocates to the nucleus, where it binds to androgen response elements (AREs) containing a 15-base pair palindromic sequence; this interaction modulates the expression of multiple androgen-dependent genes [26] (Figure 2). This is important because the action of testosterone and DHEA is mediated by AR, and this receptor is expressed in 80% of breast cancers and has been associated with reduced mortality [27]. It could therefore be used as a prognostic for the disease and as a probable target for therapy.

The number of ARs is higher in males than in females and depends on the concentration of androgens [6]. The variety of androgens and the different affinities for AR are important for understanding the regulation of the immune response since AR is found in macrophages, T lymphocytes, B cells, neutrophils, dendritic cells and NK cells [29].

## 5. AR-Independent Androgen Mechanisms of Action (Noncanonical Pathway)

Androgens also act independently of the binding of AR to DNA through noncanonical pathways, which include alternative nongenomic signaling pathways triggered by androgens via G protein-coupled receptors (GPCRs) or binding to the androgen receptor in membranes such as ZIP9, which, by activating G proteins and second messengers, increases intracellular zinc and the expression of proapoptotic genes, leading to apoptosis [30,31]. Androgen-independent signaling can also occur, which ultimately impacts androgen signaling pathways or includes an integration of nongenomic and genomic responses, as in the case of protein kinase A activation [32], or calcium signaling can occur; in fact, testosterone induces the release of intracellular calcium [33].

**Figure 3:**
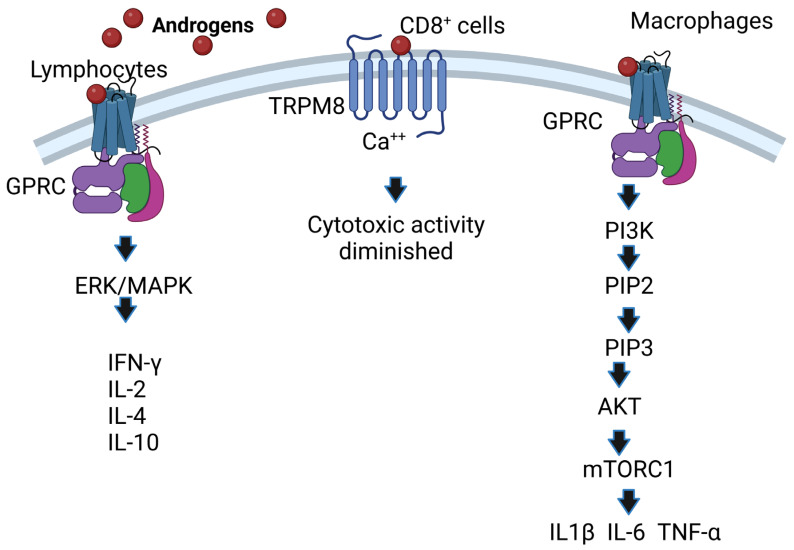
AR-independent effects of androgens. Androgens bind to membrane receptors that trigger the activation of the ERK (MAPK) pathway in lymphocytes, and IFN-γ, IL-2, IL-4 and IL-10 expression is increased. Likewise, androgens activate the phosphatidylinositol-3 kinase (PI3K) pathway; if this pathway is activated in macrophages, it regulates the expression of the cytokines IL1β, IL-6 and TNF-α. Image created based on the concepts described in [43] using the BioRender.com software. The interaction of androgens with TRPM8 causes Ca^++^ to increase, which decreases the activity of cytotoxic T cells.

Noncanonical pathways are characterized by the fact that they occur within minutes compared to the canonical pathway, which lasts for hours; it starts when androgens interact with receptors present on the plasma membrane (mAR) and affect cytosolic signaling pathways such as Src/Ras/Raf/Erk1/2 [34]; these pathways are activated by growth factors such as Src/Akt/PI3K [35]. In addition, some membrane receptors that bind testosterone are G protein-coupled receptors, and this interaction phosphorylates ErK1/2, CREB, and ATF-1 [36]. Another androgen-associated membrane receptor is TRPM8, which acts as a calcium-permeable channel [37]. Overexpression of TRPM8 decreases the cytotoxic activity of CD8^+^ T cells, which facilitates the proliferation of cancer cells [38]. Additionally, the membrane receptor GPRC6A translates the effects of androgens, and overexpression of this receptor allows HEK-293 cells lacking AR to respond to testosterone [39]. In addition, AR mutants unable to bind DNA after incubation with DHT increase the activity of protein kinase B (Akt), ERK kinases and mitogen-activated protein kinases (MAPKs) [40]. Moreover, the ERK/MAPK pathway is important because it increases the IL-2 and IFN-γ concentrations of CD8^+^ cells [41] and the expression of IL-4 and IL-10 in lymphocytes [42]. Finally, the membrane receptor GPRC in macrophages binds androgens and activates PI3K kinase, which phosphorylates inositol 3,4 diphosphate (PIP2), transforming it into inositol 3,4,5 triphosphate (PIP3). This induces the activation of Akt kinase, which in turn inhibits TSC1/2, allowing the activity of MTORC1 kinase, which phosphorylates P70S6K kinase. This enhances the expression of IL1β, IL-6 and TNF-α in macrophages [43] (Figure 3). The above is important because these cytokines are increased in response to bacterial and viral infections [44,45]. Knowledge of the mechanisms of androgen action on their target cells will contribute to understanding the relationship of androgens to susceptibility to infectious diseases, autoimmune disorders, and cancer.

## 6. Effect of Androgens on Lymphoid Organs

Several immune response cells proliferate in the spleen or mature in the thymus, and androgens regulate cell proliferation in both organs. Gonadectomy regenerates thymus size in aged male rats and increases lymphocyte proliferation, which is reversed by testosterone administration [46]. In contrast, the use of the aromatase inhibitor ATD regenerates thymus size, demonstrating the importance of testosterone biotransformation in this phenomenon [47]. In addition, testosterone promotes local glucocorticoid synthesis and release, which causes thymus involution by increasing apoptosis in the thymus tissue, and mice lacking the glucocorticoid receptor decrease thymus size [48]. This finding is important because the thymus is the organ in which bone marrow-derived T-cell precursors mature and would explain at least in part the immunosuppressive activity of testosterone in various parasitic diseases. For example, in *Trypanosoma cruzi* infections, testosterone administration causes thymus atrophy by promoting apoptosis through increased TNF-α. In contrast, DHEA administration induces thymocyte proliferation and reduces TNF-α concentration; low levels of this cytokine confer protection against this parasite. However, combined testosterone and DHEA treatment improves the immune response by reducing the number of parasites and the suppressive effects of testosterone, and by lowering TNF-α levels [3]. Furthermore, it has been described that DHEA counteracts glucocorticoid-induced thymic involution in vitro and in vivo [49]. In contrast, DHEA reduces thymus size in healthy female rats [50], confirming that the effect of DHEA is sex-dependent. Regarding other androgens, DHT increases the expression of autoimmune regulatory transcription factor (AIRE) in the thymus [8], which promotes immune tolerance and leads to a lower incidence of autoimmune diseases. DHT also decreases splenocyte proliferation in vivo [51]. In contrast, DHEA reverses the effects of corticosteroids and enhances T-lymphocyte functions by downregulating proinflammatory cytokines such as TNF-α.

## 7. Effect of Androgens on Toll-Like Receptors

Toll-like receptors (TLRs) are recognition systems relevant to the innate immune response, as they detect damage-associated molecular patterns (DAMPs) and pathogen-associated molecular patterns (PAMPs) [52]. Androgens regulate the expression of these receptors; DHEA increases the expression of TLR2 and TLR4 in macrophages of mice with sepsis, both receptors censor Gram-positive and Gram-negative bacteria, respectively [53]. However, it is not known whether DHEA modulates the expression of these TLRs in a straightforward manner. However, DHEA improves phagocytosis but does not affect TLR4 expression in neutrophils in a *Salmonella enterica* model [54]. One possible mechanism involved is that DHEA modulates the alternative splicing of TLR4, which has several spliceosomes [55], because DHEA modulates the expression of molecules such as the β-glucocorticoid receptor by this mechanism [56]. Furthermore, testosterone decreases TLR4 expression in macrophages stimulated in vitro and in lipopolysaccharide-stimulated male mice. In addition, orchectomy increases TLR4 expression [57]. In addition, the increased TLR4 expression correlates with increased AR expression in a hepatocarcinoma cell model where silencing AR with siRNA decreases the DHT-dependent increase in TLR4 [58]. In addition, testosterone increases TLR6 expression associated with the epigenetic modification caused by TLR6 promoter methylation and decreases TLR8 expression in the liver of *Plasmodium chabaudi*-infected mice, contributing to the persistence of infection [59]. On the other hand, DHT further increases TLR7 and inhibits TLR9 expression on dendritic cells by inhibiting endothelial cell apoptosis, a mechanism that is associated with the maintenance of immune tolerance [60]. These findings show that androgens suppress the expression of TLR-4, TLR-8, and TLR-9 receptors. In contrast, androgens also increase TLR4, TL-6 and TLR7 expression. These findings suggest that although the main modulatory mechanism of androgens on TLRs is the AR, there must be specific regulatory mechanisms for each cell group, which partially explains the increased susceptibility of males to a variety of infectious diseases.

## 8. Androgens Affect Cells of the Innate Immune Response

### 8.1. Macrophages, Neutrophils, and NK Cells

Macrophages, neutrophils, and NK cells constitute the first line of defense against pathogens. Macrophages and neutrophils phagocytize microorganisms and degrade them in their phagolysosomes, where reactive oxygen species (ROS) are generated by the respiratory burst [61]. Oxidizing species such as HOCl, H_2_O_2_ and OH^-^ are derived from O_2_^−^, which directly kill pathogens and promote processes such as necrosis or apoptosis [62]. In addition, the enzyme-inducible nitric oxide synthase (iNOS) synthesizes nitric oxide (NO), a reactive nitrogen species and an important mediator in the immune response; in addition, iNOS has effector and modulatory functions such as immunosuppression or cytokine response [63].

Testosterone decreases NO synthesis in macrophages in a dose-dependent manner by reducing iNOS expression [64] and increasing the intracellular free calcium concentration [65]. In addition, testosterone promotes the maturation of neutrophils [66] as well as their differentiation in vivo [67]. However, testosterone decreases the bactericidal activity of neutrophils, probably because it decreases myeloperoxidase activity and the expression of the cytokines IL-10 and TGF-β in these cells [68]. On the other hand, DHEA increases O_2_^−^ synthesis in neutrophils and macrophages [69,70]. These findings show the opposing effects of testosterone and DHEA on the oxidative activity of macrophages and neutrophils, which is important for killing pathogens.

In addition, NK cells are important in innate immunity against viral diseases and tumors because of their cytotoxic activity directly on infected or transformed cells and because they promote inflammation through the production of IFN-γ [71]. The decrease in testosterone levels because of treating men with acyline, a gonadotropin-releasing hormone (GnRH) antagonist that simulates medical castration, increases the proliferation of NK cells [72]. In contrast, DHEA positively modulates immunity by increasing the cytotoxic activity of NK cells on K562 tumor cells in vitro by increasing the synthesis of insulin-like growth factor-I (IGF-I); it can induce autocrine and paracrine control of immune cell replication and function [73]. Furthermore, when NK cells are stimulated with IGF-1, this factor binds to its receptor on NK cells, increases the expression of the transcription factor NFIL-3 and promotes the expression of CD69 (a mediator of NK-cell cytotoxic activity) and genes encoding perforins and granzyme B [74]. These findings show that testosterone and DHEA modulate the activity and proliferation of dendritic cells, neutrophils, and NK cells in a different manner, which is important for the resolution of infectious diseases.

### 8.2. Dendritic Cells

Dendritic cells are specialists in immune surveillance, antigen capture, antigen processing and presentation to T cells; this interaction promotes the maturation of naive T cells into Th1 or Th2 lymphocytes, which is indispensable for the development of the adaptive immune response. In addition, dendritic cells in men secrete a lower concentration of IFN-α than dendritic cells from women during viral infections [75], suggesting a modulatory effect of androgens. In addition, lowering the androgen concentration in mice by castration promotes the maturation of dendritic cells [76].

Androgens modulate cytokine synthesis in dendritic cells. A clinical study in men with type 2 diabetes who developed partial testosterone deficiency showed testosterone immunosuppressive activity in reducing the synthesis of the proinflammatory cytokines IL-1β, IL-6 and TNF-α, an effect that persists after termination of testosterone treatment. In addition, dendritic cells from men with hypogonadism show increased activation of their dendritic cells inversely related to testosterone concentration, which may at least partially explain the immunosuppressive and anti-inflammatory effects of testosterone [77]. In contrast, DHEA and DHEA-S induce maturation and increase the ability of dendritic cells to activate Th1 lymphocytes to synthesize IFN-γ and IL-4 in pregnant females [78]. Furthermore, DHEA promotes the maturation of monocytes into dendritic cells and antagonizes the effect of other adrenal hormones, such as corticosteroids [79]. Interestingly, DHT does not modify dendritic cell differentiation in vitro [80]. This provides evidence of opposite effects of androgens on dendritic cells.

## 9. Effects of Androgens on Cells of the Adaptive Immune Response

### 9.1. Th1 and Th2 Lymphocytes

Lymphocytes are extremely important cells in the immune response; they synthesize cytokines that modulate the differentiation, proliferation, activation, and secretion of molecules in cells of the immune system. Th1 lymphocytes produce proinflammatory cytokines such as IFN-γ and TNF-α that promote innate and adaptive immune responses against intracellular pathogens and have antitumor effects [81,82]. On the other hand, Th2 cells promote IgG1 and IgE production important against extracellular pathogens such as helminths [83]. Therefore, it is important to understand the effects of androgens on these lymphocyte populations.

Decreasing the concentration of androgens by castration lowers the threshold response to IL-12 and increases the expression of T-bet (a transcription factor that promotes the differentiation of CD4^+^ T cells to a proinflammatory Th1 profile) and Stat4 phosphorylation [84]. Stat4 once phosphorylated is a mediator of inflammation, and dysregulation of STAT 4 is associated with the development of autoimmune diseases and cancer [85]. In contrast, testosterone administration to mice negatively regulates differentiation to Th1 cells by inhibiting IL-12-induced Stat4 phosphorylation; in this mechanism, AR binds to the phosphatase Ptpn1 and consequently inhibits IL-12 signalling in CD4^+^ cells [86]. Moreover, testosterone acts directly via interaction with AR on CD4^+^ cells to upregulate IL-10 expression, an anti-inflammatory cytokine that promotes a Th2-type response [87]. In addition, to modulate the Th1/Th2 balance, testosterone promotes thymocyte apoptosis by enhancing the expression of FAS and caspase-8 [88]. In contrast, DHEA stimulates the activation of Th1 lymphocytes by increasing dendritic cell maturation and activity in vitro [89]. DHEA also induces thymocyte proliferation during *Trypanosoma cruzi* infections [3]. Moreover, DHEA decreases Th2-type responses independent of cortisol concentration in individuals with atopic dermatitis [90]. These findings reveal that testosterone and DHEA modulate the balance of Th1 and Th2 responses differently; testosterone stimulates the proliferation of Th2 cells, whereas DHEA promotes the maturation to Th1 cells.

### 9.2. Th17 and Regulatory T Cells

Regulatory T cells are involved in immune tolerance and regulate the proinflammatory response through the synthesis of IL-10 and TGF-β. Th17 lymphocytes are involved in regulating the balance of the proinflammatory and anti-inflammatory response, which is important in the defense against intracellular parasites [91]. However, Th17 lymphocytes are also associated with a chronic proinflammatory response. In addition, Th17 lymphocytes in males are lower in number than in females, which is associated with the lower susceptibility to rheumatoid arthritis in males [92]. It is therefore possible that androgens are involved in this phenomenon, as testosterone decreases Th17-cell differentiation in vitro [93]. Furthermore, DHEA indirectly decreases the percentage of Th17 cells by inducing IL-10 secretion in regulatory T cells [94]. In addition, both testosterone and DHEA increase the expression of the transcription factor Foxp3, which is necessary for the differentiation of T lymphocytes to regulatory T lymphocytes [95]. This makes sense because men have a higher number of regulatory T lymphocytes and less development of autoimmune diseases than women [96]. This suggests that androgens are necessary for the maintenance of immune tolerance by modulating the number of Th17 and regulatory T lymphocytes.

### 9.3. Cytotoxic T-Lymphocytes

CD8^+^ lymphocytes recognize virus-infected cells and intracellular pathogens and eliminate them and produce IFN-γ that activates macrophages. Androgens negatively modulate CD8^+^ cell function; for example, when the androgen concentration is reduced by gonadectomy, this cell population increases in peripheral lymphoid tissues of C57Bl/6 mice; furthermore, in vitro CD8^+^ stimulation with CD28 increases the number of CD8^+^ cells [97]. In addition, exogenous administration of testosterone to gonadectomized male mice infected with influenza A virus decreases the number and activity of CD8^+^ lymphocytes by binding to the AR. In contrast, the administration of DHEA decreases the severity of influenza A virus infection in these mice [98]. Furthermore, in a murine model of trauma and sepsis achieved by cecal ligation and puncture, DHEA administration preserves T CD8^+^ lymphocyte activity at normal levels [99]. Further evidence for the immunoprotective activity of DHEA on CD8^+^ lymphocytes is that dexamethasone-immunosuppressed mice infected with *Cryptosporidium parvum* and treated with DHEA had increased CD4^+^ and CD8^+^ populations [100]. The above is evidence of the opposite effects of testosterone and DHEA on CD8^+^ lymphocytes.

### 9.4. B Lymphocytes

B cells are important for the adaptive immune response, as they develop into antibody-producing cells that are indispensable for killing numerous pathogens, such as bacteria, viruses, and parasites [101,102].

Lowering testosterone levels by castration of C57BL/6N mice increases the proliferation of B-cell precursor cells in the bone marrow, and administration of testosterone reverses this effect. Given that ARs have not been detected in mature B cells but have been detected in both B-cell precursors and bone marrow stromal cells, it is likely that testosterone is involved in the maturation of B cells [103]. One possible explanation for this finding is that testosterone decreases the concentration of B-cell activating factor (BAFF), which is a survival factor for B cells. In addition, AR knockout mice and gonadectomized male mice have a higher concentration of BAFF than normal individuals [104]. On the other hand, peripheral blood cells incubated with testosterone decrease the concentration of IL-6, which leads to a reduction in the concentration of IgG and IgM antibodies in vitro in a dose-dependent manner and independent of B-lymphocyte proliferation, suggesting that testosterone negatively modulates B-lymphocyte activity [105].

In addition, administration of testosterone to mice infected with *Plasmodium chabaudi* reduces the concentration of antibodies for several weeks, which increases the susceptibility to this parasite [1]. Conversely, when human B-lymphocytes are treated with physiological concentrations of DHEA (1 × 10^−6^ M to 1 × 10^−7^ M), the proliferation of human B-lymphocytes activated with *Staphylococcus aureus* increases in vitro [106]. In contrast, when DHEA was added at higher concentrations (5 × 10^−5^ M and 1 × 10^−4^ M), the number of B cells decreased [106]. These findings reveal that testosterone and DHEA induce different effects on B-cell activity in a concentration-dependent manner.

The analysis of the effects of testosterone and DHEA on the most important immune response cells is summarized in Table 1. This knowledge is of particular interest to understand sexual dimorphism in infectious and autoimmune diseases.

## 10. Effect of Androgens on the Cytokines IFN-γ, TNF-α, IL-2, IL-10, TGF-B, IL-4, IL-5, IL-6, and IL-17

Cytokines are key molecules in the modulation and communication of immune response cells. Androgens have been reported to have anti-inflammatory properties, which is confirmed by the fact that castration causes an inflammatory state [108]. In addition, induction of hypogonadism in young men by means of GnRH agonists increases TNF-α and IL-1β [109]. Hypogonadism also increases IL-10 [110]. Moreover, testosterone replacement therapy decreases the levels of proinflammatory cytokines. Testosterone replacement therapy to testosterone-deficient men undergoing type 2 diabetes decreases the synthesis of the proinflammatory cytokines IL-1β, IL-6 and TNF-α in antigen-presenting cells [77]. In general, testosterone negatively modulates the production of IFN-γ, TNF-α, IL-2, and IL-6, which are distinctive from the inflammatory response. In contrast, DHEA increases IL-2 production; lymphocytes from DHEA-treated mice increase the IL-2 concentration, and lymphocytes from normal mice cultured in the presence of DHEA increase the IL-2 concentration [111]. Furthermore, during infection with *Trypanosoma cruzi*, DHEA increases the concentration of IFN-γ [112]. Rats infected with this parasite have a higher concentration of corticosterone than DHEA, and this difference is associated with elevated TNF-α and decreased IL-10 levels, which aggravate the disease [113]. DHEA has been suggested to promote the Th1 response by increasing Ca^+^ ATPase activity and decreasing Na^+^ K^+^ ATPase activity [106], and this enzyme is required for the activation and proliferation of lymphocytes [114]. Additionally, DHEA at physiological and pharmacological concentrations (5 × 10^−9^ to 5 × 10^−6^) reduces the expression of TNF-α and IL-6 by macrophages [115].

However, IL-4 and IL-5 are features of the Th2 response, and DHEA reduces IL-4 concentrations in vitro and in vivo [90,116]. In addition, DHEA suppresses the IL-5 concentration in a dose-dependent manner; therefore, DHEA favors the Th1 response. In contrast, elevated testosterone concentrations promote IL-5 and, therefore, the Th2 response [117]. Regulatory T cells synthesize IL-10 and TGF-β, both with anti-inflammatory properties. IL-10 regulates the proinflammatory response and decreases the severity of pathology in parasitic infections [118], and TGF-β modulates lymphocyte and macrophage proliferation [119]. Interestingly, DHEA and testosterone increase the plasma levels of IL-10 and TGF-β [68,95,120]. One possible explanation for this finding is that androgens increase Foxp3 expression, which results in the differentiation of TCD4^+^ lymphocytes to regulatory T cells that synthesize IL-10 and TGF-β [95]. This would explain why androgens promote immune tolerance.

On the other hand, the cytokine IL-17 participates in the defense against infections by bacteria, parasites, fungi and viruses [121]; IL-17 is involved in the development of inflammatory diseases, an example of which is multiple sclerosis [122]. DHEA or testosterone administration decreases IL-17 levels as well as the expression of RORC2, a key transcription factor for Th-17 cells [123].

The above demonstrates the opposite effects of testosterone and DHEA on the regulation of inflammatory responses through cytokine production. Testosterone downregulates the production of proinflammatory cytokines such as IL-1β, TNF-α, IL-6, and IL-17, while DHEA upregulates IFN-γ and IL-2 production. However, both hormones increase IL-10 and TGF-β but decrease IL-17. The effects of androgens on the concentrations of the most important cytokines are summarized in Table 2.

Interestingly, DHEA is recognized as an anti-glucocorticoid hormone [124], and DHEA counteracts the effects of cortisol on the expression of RACK1, an adaptor protein that interacts with different PKC isoforms, and is required for immune response cells and PKC-dependent signaling pathways to function properly [125]. In addition, RACK1 modulates cortisol inhibition of LPS-induced cytokine release. DHEA positively regulates the mRNA expression of some components of spliceosome-localized serine/arginine-rich proteins (SRp) that are key regulators of alternative splicing of the β-glucocorticoid receptor gene (GRβ) [125]. Silencing GRβ expression with small interfering RNA blocks the effect of DHEA on RACK1 and thus does not release the cytokines induced by LPS stimulation. This is important because it suggests that the activity of spliceosome proteins involved in alternative GRβ mRNA splicing could constitute a therapeutic target for regulating glucocorticoid activities in the immune system.

## 11. Conclusions

**Figure 4:**
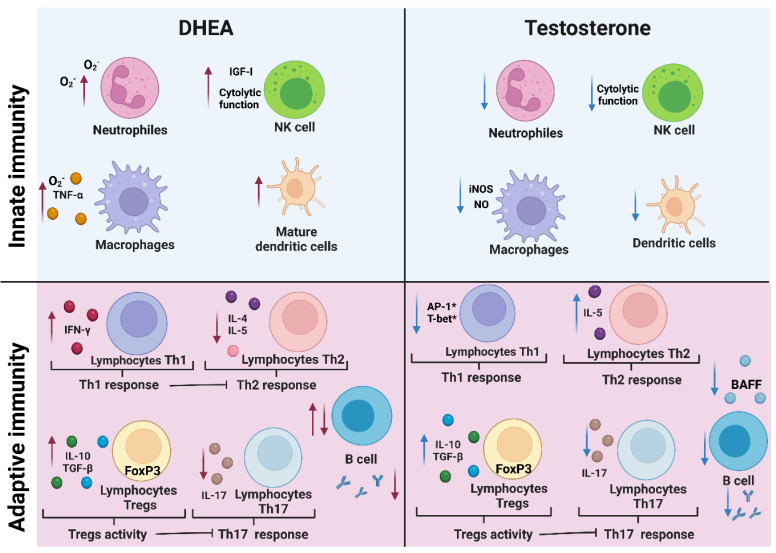
Effect of DHEA and testosterone on cells of the innate and adaptive immune response. Androgens modulate the proliferation of immune cells, the concentration of cytokines and the expression of transcription factors that modulate the immune response. The effects of DHEA are represented by red arrows, and those of testosterone are represented by blue arrows. The asterisk (*) indicates transcription factors probably involved in the mechanism of action. During the innate immune response, DHEA increases O_2_^−^ synthesis in neutrophils and macrophages, and the cytotoxic activity of NK cells by enhancing the secretion of insulin-like growth factor-I (IGF-1). Furthermore, DHEA promotes the maturation of dendritic cells. In contrast, testosterone decreases neutrophil proliferation, NK-cell cytolytic activity, and macrophage nitric oxide (NO) synthesis by decreasing iNOS expression and dendritic cell proliferation.

Androgens modulate several aspects of the immune response such as immune cell proliferation, cytokine secretion and Toll-like receptor expression. However, testosterone has immunosuppressive properties, whereas DHEA promotes the Th1 response (Figure 4). Interestingly, both androgens stimulate immune tolerance by inducing the differentiation of naive T lymphocytes to regulatory T lymphocytes, which in turn decreases the Th17 response. The importance of androgens on the immune response is evident; this justifies their study, since it would facilitate understanding the sexual dimorphism that occurs in infectious and autoimmune diseases. Furthermore, given that androgens are the main inducers of prostate tumor growth and that the function of these steroids depends on interaction with their receptor, the use of drugs that prevent, compete with or modify the conformation of the androgen receptor could be used as therapies to inhibit the expression of genes involved in androgen-dependent cancer cell proliferation.

In the adaptive response, DHEA increases the Th1 response by increasing IFN-γ synthesis, which in turn suppresses the Th2 response. DHEA also increases the proliferation of regulatory T cells by enhancing the expression of FoxP3, whose activity suppresses the Th17 response. In addition, DHEA negatively and positively modulates B-cell proliferation depending on its concentration. Conversely, testosterone decreases the Th1 response, probably because it suppresses the expression of T-bet, which is the master modulator of the Th1 response. Testosterone also promotes the Th2-type response; it increases the activity of regulatory T lymphocytes and the secretion of IL-10 and TGF-β through increased expression of FoxP3, which in turn suppresses the Th17 response. In addition, testosterone decreases B-cell proliferation by reducing the concentration of B-cell survival factor (BAFF), which decreases the concentration of antibodies. Figure created with BioRender.com.

## Figures and Tables

**Table 1:** Effect of testosterone and DHEA on innate and adaptive immune response cells.

Type	Cell	Testosterone	REF	DHEA	REF
Innate	Macrophages	↓ the secretion of NO	[64]	↑ synthesis of O_2_^−^	[70]
	NK cells	↓ proliferation	[72]	↑ cytotoxic activity	[73]
	Neutrophis	↓ bactericidal activity	[68]	↑ synthesis of O_2_^−^	[69]
	Dendritic cells	↓ maturation	[76]	↑ maduration	[78]
Adaptive	Th1 lymphocytes	↓ the expression of T-bet	[84]	↑ activation	[89]
	Th2 lymphocytes	is favored by the suppression of IL-12	[87]	↓ activation	[90]
	Regulatory T lymphocytes	↑ the expression of Foxp3	[95]	↑ the expression of Foxp3	[95]
	Th17 lymphocytes	↓ proliferation	[93]	↓ proliferation	[94]
	B cells	↓ proliferation and antibody secretion	[103]	modulates their proliferation	[106,107]

↓ represents decrease and ↑ increase.

**Table 2:** Effect of androgens on cytokines.

Cytokine	Function	Testosterone (Reference)	DHT (Reference)	DHEA (Reference)
IFN-γ	Lymphocyte and macrophage activation.	does not change [116]	↑ [116]	↑ [112]
TNF-α	Proinflamatory response, macrophage activation	Inhibits its effects [126]	Inhibits its effects [127]	↓ [115]
IL-2	Lymphocyte activation	↓ [128]	↑ [129]	↑ [106]
IL-10	Antiinflamatory response, immunological tolerance	↑ [95]	↑ [87]	↑ [68]
TGF-β	Antiinflamatory response	↑ [120]	↑ [129]	↑ [120]
IL-4	Th2 response	- [116]	↓ [116]	↓ [90]
IL-5	Antibody secretion	↑ [117]	↓ [116]	↓ [130]
IL-6	B cell differentiation	↓ [128]	↓ [127]	↓ [115]
IL-17	Chronic proinflammatory response	↓ [123]	↓ [131]	↓ [123]

↓ represents decrease and ↑ increase.

## Data Availability

Not applicable.
